# Clinical efficacy observation and safety evaluation of acupuncture for intractable facial paralysis: a single-blinded randomized controlled pilot trial

**DOI:** 10.3389/fneur.2025.1504089

**Published:** 2025-10-16

**Authors:** Hongyu Xie, Yu Xia, Lele Zhang, Aihong Yuan, Jun Yang, Ling Cheng, Ting Gao, Bo Li, Xuwen Yuan, Min Ye, Wenjing Kan, Jie Shi, Zhen Liu, Fei Yuan, Chao Zhou, Xiaojun Liu

**Affiliations:** ^1^Second Department of Acupuncture Rehabilitation, The First Affiliated Hospital of Anhui University of Chinese Medicine, Hefei, China; ^2^Anhui University of Chinese Medicine, Hefei, China; ^3^Fourth Department of Geriatric Diseases, The Second Affiliated Hospital of Anhui University of Chinese Medicine, Hefei, China; ^4^Second Department of Acupuncture Rehabilitation, Shuguang Hospital Anhui Branch Affiliated to Shanghai University of Traditional Chinese Medicine, Hefei, China; ^5^Department of Acupuncture, Bozhou Hospital of Traditional Chinese Medicine, Bozhou, China; ^6^Department of Traditional Chinese Medicine, First Affiliated Hospital of Anhui Medical University, Hefei, China; ^7^Bozhou Vocational and Technical College, Bozhou, China

**Keywords:** acupuncture, intractable facial paralysis, Anzhong Facial Paralysis Precision Scale (Oral Commissure Ptosis Grading Scale), surface electromyography, randomized controlled pilot trial

## Abstract

**Introduction:**

Acupuncture can effectively improve the clinical symptoms of intractable facial paralysis (IFP). However, it is not yet clear whether the use of acupuncture manipulation affects the therapeutic effect. This study aims to conduct a preliminary exploration of the clinical efficacy and safety of acupuncture treatment for IFP using acupuncture manipulation.

**Methods:**

A single-center, single-blind, randomized controlled pilot trial was conducted from December 2022 to December 2023, involving 40 IFP participants, divided into the ordinary acupuncture group (OAG, *n* = 20) and the characteristic acupuncture group (CAG, *n* = 20). The OAG underwent a standardized acupuncture protocol comprising 3 weekly sessions over a 10-week period. This structured regimen included 3 consecutive treatment cycles (10 sessions/cycle), culminating in 30 total therapeutic interventions. The CAG has performed characteristic acupuncture manipulation on this basis, with the same frequency and duration of treatment as the OAG. Assess the patient’s facial recovery status at baseline and after 10, 20, and 30 treatments.

**Results:**

After the second treatment course, the difference in the Anzhong Facial Paralysis Precision Scale (Oral Commissure Ptosis Grading Scale) levels between the two groups began to show statistical significance (*p* < 0.05); After the third treatment course, the scores changes in Sunnybrook Facial Grading System (SFGS) scale between the two groups began to show statistical significance (*p* < 0.05); After the first treatment course, there was statistical significance in the average ratio changes at LI20 (Yingxiang) and ST4 (Dicang) between the two groups (*p* < 0.05). After the second treatment course, statistical significance in the average ratio changes was observed at GB14 (Yangbai), SI18 (Quanliao), LI20 (Yingxiang), and ST4 (Dicang) between the two groups (*p* < 0.05). After the third treatment course, statistical significance in the average ratio changes was found at all acupoints between the two groups (*p* < 0.05). Consequently, the CAG group demonstrated superior therapeutic efficacy compared to the OAG group.

**Conclusion:**

Acupuncture has a good therapeutic effect on IFP, and the combination of characteristic acupuncture manipulation has a better therapeutic effect. However, these conclusions require further validation in larger clinical studies.

**Clinical trial registration:**

https://www.chictr.org.cn/, Identifier ChiCTR2200065442.

## Introduction

Peripheral facial paralysis is the facial nerve paralysis caused by the injured facial nerve nucleus and the parts below the nucleus. Its clinical characteristics are mainly the dysfunction of the motor function of the facial expression muscles dominated by the injured facial nerve, which is mainly manifested in the weakening or disappearance of the facial expression action on the affected side, accompanied by mouth deviation, oral commissure salivation, eyebrow lifting limitation, and inability to close eyes. It is the most common functional disorder of facial expression muscles in clinical practice (1), affecting appearance and causing dual physical and psychological harm to patients. According to epidemiology (2), it occurs frequently in spring and winter, with an incidence rate of 11.5–55.3/100000, and has obvious geographical and seasonal distribution. This disease is the fifth common disease in neurology. The pathogenesis of peripheral facial paralysis is currently not fully understood. It is generally believed to be a non-specific inflammatory lesion of the facial nerve caused by the combined action of multiple pathogenic factors (3, 4). It can be caused by viral infection, abnormal local anatomical structure, immune abnormalities, environmental damage, and other pathogenic factors (5), leading to local microcirculatory disorders, ischemia and hypoxia, and promoting facial nerve bleeding, edema, and demyelination changes, thereby causing facial nerve dysfunction and facial nerve distribution area paralysis. Modern medicine mainly uses antiviral, glucocorticoid, vitamin, nutritional nerve drugs, and circulation improving drugs to relieve facial nerve compression, avoid facial nerve degeneration, and promote the recovery of facial nerve function.

According to domestic and foreign research reports (6), more than 10% of patients still have serious sequelae after treatment. At present, there is no unified definition of Intractable Facial Paralysis (IFP) in clinical practice. Most clinical studies categorize patients based on the duration of their illness, and define peripheral facial paralysis caused by unknown reasons for more than 3 months (including “Bell’s palsy” and “Hunter’s syndrome”) as IFP (7). However, modern medicine does not have effective treatments for patients with peripheral facial paralysis who have progressed to this stage, and those with a longer course of illness are prone to a series of residual symptoms such as “inversion” (8). The manifestation of “inversion” is muscle spasm on the affected side, where the oral commissure is pulled toward the affected side due to muscle fiber contraction and twitching. There are two reasons for this: firstly, due to the prolonged course of the disease, the paralyzed facial nerve function has not been restored, and the paralyzed muscles lose nerve innervation, thus atrophy occurs; The second is the phenomenon of “false inversion,” which is caused by the loss or mistreatment of the affected side muscles, resulting in atrophy and spasms of the affected side muscles, leading to the reversal of the oral commissure toward the affected side (9). The phenomenon of facial paralysis “inversion” is speculated to be related to demyelination and abnormal growth of nerves based on existing clinical research (10).

Acupuncture has a long history of treating facial paralysis, with definite curative effect, and facial paralysis is one of the dominant diseases in acupuncture treatment. As early as the 1970s, the World Health Organization recognized acupuncture was suitable for the treatment of this disease (11), and this treatment is widely used to treat facial paralysis in recovery and sequelae stages. Clinical studies have confirmed that acupuncture has a significant effect on IFP (12–14), but there are still some problems such as the lack of unified treatment methods and incomplete evaluation standards of efficacy. Mechanistic studies have shown that in pathological conditions, patients with IFP exhibit abnormal functional connectivity in brain regions. HE X et al. (15) pointed out that acupuncture modulates the functional connectivity of the brain’s default mode network and related areas, thereby regulating internal homeostasis. Research by HU S et al. (16) demonstrated that the functional connectivity between the anterior cingulate cortex on one side and the primary motor cortex, supplementary motor area, premotor area, as well as the bilateral primary sensory cortex and dorsolateral prefrontal cortex, is enhanced and positively correlated with time. These regions are associated with the movement and control of facial expression muscles and proprioceptive sensations. The team’s early research (17, 18) confirmed the idea of applying acupuncture to treat facial paralysis in the whole process, and confirmed that acupuncture intervention still had curative effect in the sequelae stage. Also functional magnetic resonance technology was used to determine that acupuncture treatment could promote the reorganization of the cortical function of facial paralysis patients. It was the first time of our team to find different changes in the response of the brains of facial paralysis patients to acupuncture at different pathological stages, and acupuncture promoted the cortical functional recombination pattern network in patients with facial palsy with long disease course, which partially revealed the neurobiological mechanism of acupuncture treatment of facial paralysis.

**Figure 1:**
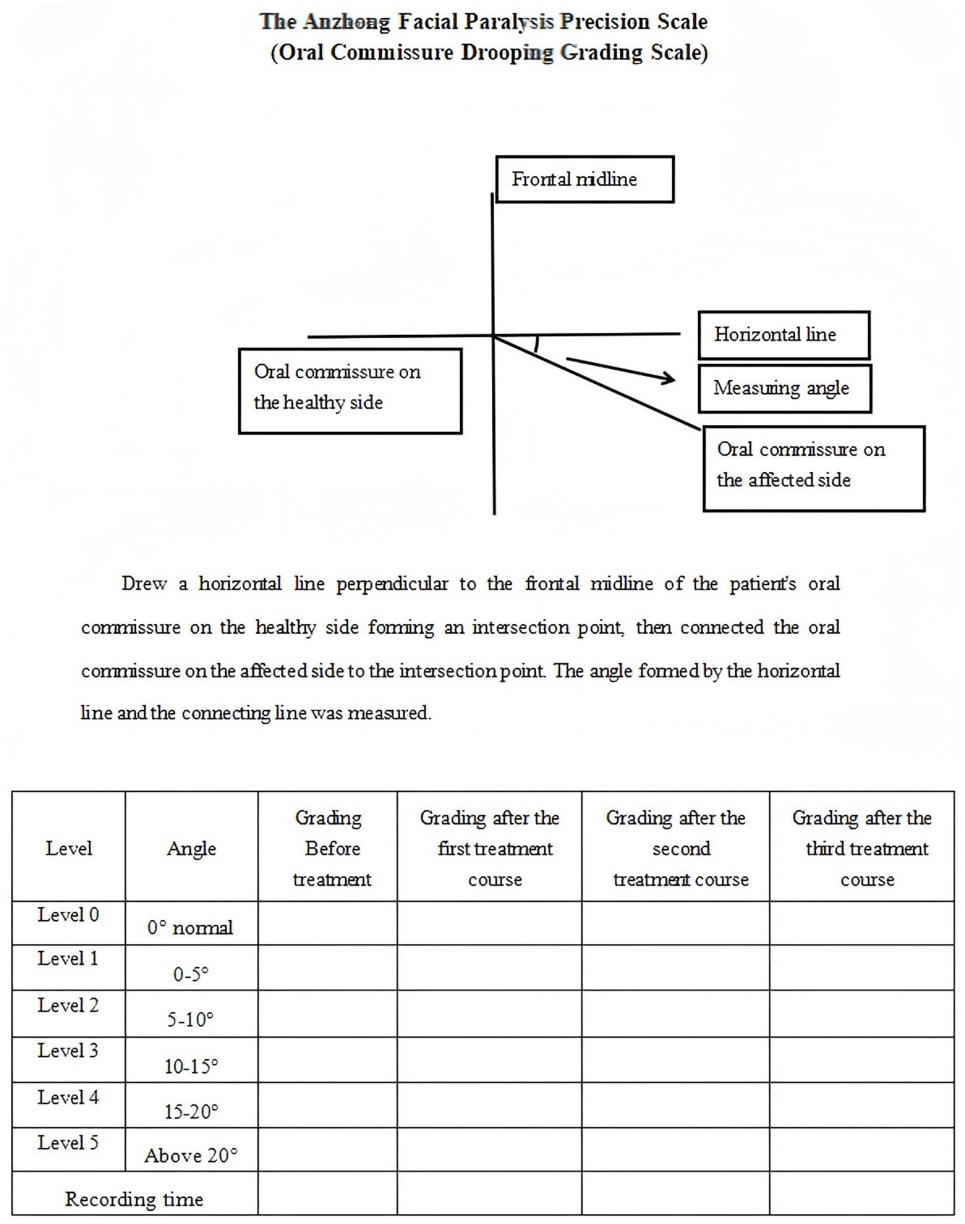
The Anzhong Facial Paralysis Precision Scale (Oral Commissure Ptosis Grading Scale).

Therefore, this study takes IFP patients as the research object, acupuncture as the main treatment method, and compares characteristic acupuncture with ordinary acupuncture. Characteristic acupuncture uses three characteristic acupuncture manipulation, namely, “long needle penetration acupuncture, stagnating needle lifting and pulling acupuncture, healthy side balance acupuncture” (19). By combining the Anzhong Facial Paralysis Precision Scale (Oral Commissure Ptosis Grading Scale) (as shown in Figure 1) produced by our team, the Sunnybrook Facial Grading System (SFGS) scale, and modern instrument surface electromyography (sEMG), we aimed to evaluate the efficacy, safety, and superiority of acupuncture manipulation in treating IFP, and verify the sensitivity and advantages of Anzhong Facial Paralysis Precision Scale (Oral Commissure Ptosis Grading Scale) in evaluating IFP. The ultimate goal was to establish a novel approach and perspective for diagnosing, treating, and evaluating the disease, in order to enhance the clinical recovery rate of IFP and offer a scientific foundation for acupuncture interventions.

## Methods

### Design and ethical approval

This study was conducted between December 2022 and December 2023 at the First Affiliated Hospital of Anhui University of Chinese Medicine, and was designed as a 10-week, single-center, single-blind, randomized controlled pilot trial. The primary objective was to evaluate the effects of acupuncture manipulation on the recovery of facial muscle and nerve function in IFP patients. The study protocol adhered to the Standard Protocol Items: Recommendations for Interventional Trials (SPIRIT) guidelines and followed the principles outlined in the Consolidated Standards of Reporting Trials (CONSORT) and the Standards for Reporting Interventions in Clinical Trials of Acupuncture (STRICTA) (20). Approval for the study was granted by the Institutional Review Committee of the First Affiliated Hospital of Anhui University of Chinese Medicine (Approval No.: 2022AH-79). Additionally, the trial was registered at the China Clinical Trial Registry (Registration No.: ChiCTR2200065442, Protocol Version No.: V1.0).

### Participants

Recruitment strategy: IFP patients were recruited from the outpatient or the ward of the Second Department of Acupuncture Rehabilitation and the Department of Neurology in the First Affiliated Hospital of Anhui University of Chinese Medicine. In addition, patients were recruited from the community through posting notices, online publishing, and other means. Patients who agreed to participate in this study were diagnosed by our department’s experts and neurology specialists. Eligible patients were screened by researchers based on inclusion and exclusion criteria.

The inclusion criteria referring to the diagnosis of facial paralysis in Newly Compiled Modern Practical Internal Medicine (2014) were: (1) Medical history: a history of viral infection, facial cooling, and hair blowing; (2) Clinical manifestations: sudden onset, unilateral facial discomfort in patients, progressing to complete facial paralysis within 1–2 h of onset, deflection of angle of mouth, disappearance of forehead wrinkles and nasolabial folds, inability to lift eyebrows, close eyes, and show teeth, and some patients may initially have symptoms such as pain in the mastoid process inside and behind the ear. (3) Other examinations: No other positive signs of the nervous system were found; (4) Course of illness: failure to heal for ≥ 3 months.

The exclusion criteria were: (1) Other diseases (such as Guillain Barre syndrome, traumatic brain injury, intracranial tumors, labyrinthitis, etc.) that cause facial paralysis or facial rupture infections; (2) Breastfeeding, pregnancy, and pre pregnancy women; (3) Any system with serious primary illness or mental disorders cannot cooperate with treatment; (4) Currently receiving treatment from other traditional Chinese and Western medicine; (5) Dizziness, intolerance, and adherence to treatment; (6) During the treatment period, if there are other sudden illnesses that require immediate treatment.

### Study procedures

The study population comprised 51 patients with IFP who were recruited at the study site. In total, 43 participants were enlisted, while only 40 IFP patients completed a 3-course study. The designated researchers who did not participate in the trial were responsible for recruiting participants, while another independent researcher who did not participate in treatment and recruitment generated a random allocation sequence. The generated random numbers were placed in opaque sealed envelopes using a random number table, and envelopes were randomly selected in the order of the patients’ medical visits. The participants were divided into the characteristic acupuncture group (CAG, *n* = 20) and the ordinary acupuncture group (OAG, *n* = 20) in a 1:1 ratio. In this study, patients, outcome assessors, and statistical analysts were blinded to the treatment, and patients were treated in separate rooms to reduce communication. Due to the particularity of acupuncture manipulation, it is impossible to achieve blindness for the operator.

Adverse events occurring during the study period were systematically documented in the Case Report Form. The recorded information encompassed the timing, cause, clinical symptoms, signs, and corresponding emergency response plans for each adverse event. Additionally, any laboratory test abnormalities deemed clinically significant were also included. Furthermore, clinical doctors evaluated all events to determine their relevance and severity in relation to the intervention measures.

### Interventions

In this study, the disposable acupuncture needles’ size were 1.5 inch (0.25 mm × 40 mm) and 1.0 inch (0.25 mm × 25 mm) (Huatuo, Suzhou Medical Supplies Factory Co., Ltd.).

### OAG

**Figure 2:**
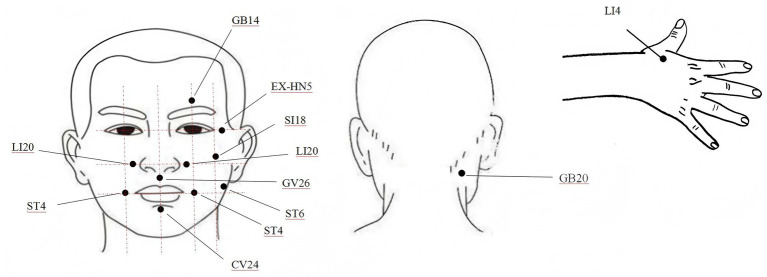
Acupoints diagrams.

Patients took a sitting position, and the local skin of acupoints were routinely disinfected and punctured. The main acupoints selected were the head and face acupoints, including GB14 (Yangbai), SI18 (Quanliao), LI20 (Yingxiang), GV26 (Shuigou), ST4 (Dicang), CV24 (Chengjiang), EX-HN5 (Taiyang), ST6 (Jiache), GB20 (Fengchi), and LI4 (Hegu), as shown in Figure 2.

### CAG

The CAG has performed characteristic acupuncture manipulation on the basis of the OAG, and the acupuncture treatment was carried out by acupuncturists who were professionally qualified for more than 5 years. Manipulation: Each patient was seated upright, and the local skin of the acupoints was routinely disinfected, mainly using characteristic acupuncture manipulation such as long needle penetration acupuncture, stagnating needle lifting and pulling acupuncture, healthy side balance acupuncture. Long needle penetration acupuncture: inserting the 0.25 mm × 40 mm acupuncture needle into the subcutaneous area of the acupoint at a 15 degree angle to the skin, and then press it tightly against the subcutaneous area of the face and pierce toward another acupoint. Stagnating needle lifting and pulling acupuncture: the 0.25 mm × 40 mm acupuncture needle is inserted paralleled to the face into acupoints and muscles to a certain depth, then twisting the needle handle to wrap the needle around the muscle fibers, driving the facial muscles to lift upwards several times. Healthy side balance acupuncture: in addition to the normal acupuncture treatment on the affected side, the acupoints were properly selected on the healthy side, and the acupuncture needles of 0.25 mm × 25 mm in the middle were used for shallow needling (5 mm). The dose of stimulation was as small as to the degree of local slight needling sensation.

**Figure 3:**
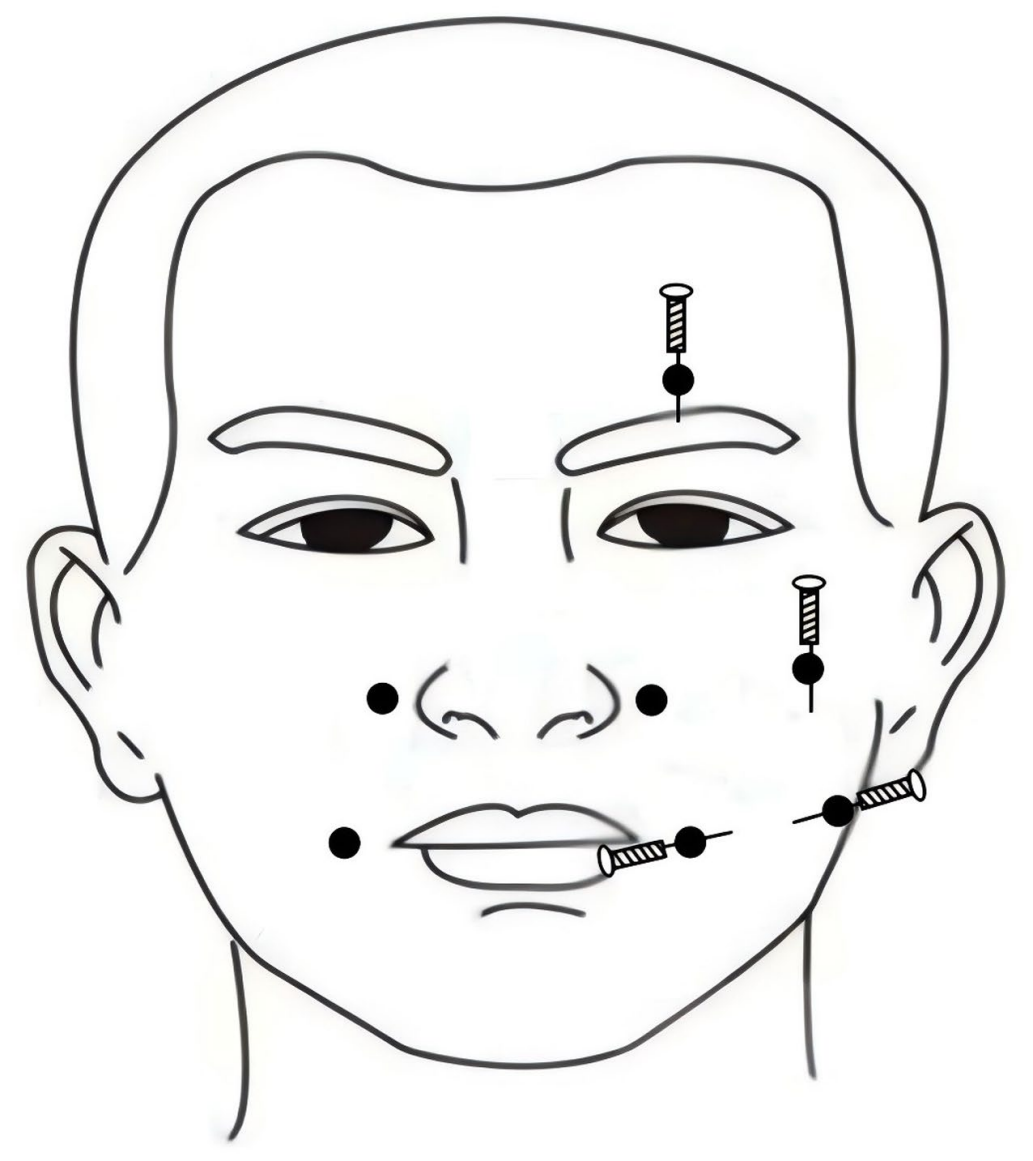
Acupuncture manipulation diagram.

The affected side’s ST4 (Dicang) and ST6 (Jiache) were penetrated through one to the other, and the long needle penetration acupuncture was used. The needle tips were needled into GB14 (Yangbai) and SI18 (Quanliao) downwards, and rotated in one direction, and then the needle handle was pulled after the stagnant sensation of needling, performing the stagnating needle lifting and pulling acupuncture. ST4 (Dicang) and LI20 (Yingxiang) were shallow needled, performing healthy side balance acupuncture. Routine needling was performed on the remaining acupoints, as shown in Figure 3.

Both groups received acupuncture treatment for 30 min per session, following a standardized protocol consisting of 3 weekly sessions over a 10-week period. This structured regimen included 3 consecutive treatment cycles (10 sessions/cycle), culminating in 30 total therapeutic interventions.

During the study, patients were allowed to use neurotrophic medications, and required to report to the researchers whether taking any other medication(s), and record the name, dosage, and date of the medication(s) used.

### Observation indicators

#### Main observation indicator

According to the Anzhong Facial Paralysis Precision Scale (Oral Commissure Ptosis Grading Scale), we drew a horizontal line perpendicular to the frontal midline of the patient’s oral commissure on the healthy side forming an intersection point, then connected the oral commissure on the affected side to the intersection point. The angle formed by the horizontal line and the connecting line was measured and analyzed. When the angle approached 0, it was considered normal. It was divided into four levels, 0–5. The lower the level, the less skewed oral commissure, and the better the recovery.

#### Secondary observation indicators

1. According to SFGS scale, supplementary evaluation was conducted from three aspects: static, voluntary movement, and linkage of facial muscles in patients. Scores ranged from 0 to 100, with higher scores indicating better recovery of facial nerve function.
2. Root mean square (RMS) average ratio: The sEMG analysis system Bio Neuro Infiniti Flexcomp software version 6.0 was used to detect the RMS values of the muscle groups at the locations of the patients’ affected and healthy acupoints, including GB14 (Yangbai), SI18 (Quanliao), ST6 (Jiache), LI20 (Yingxiang), and ST4 (Dicang). The RMS average ratio of sEMG on the affected and healthy sides was compared. The larger the ratio, the stronger the facial muscle strength, and the better the degree of recovery.

All patients were evaluated using the above scales and examinations before the first treatment, after the 10th, 20th, and 30th treatments. The researchers conducting the evaluation were doctors from our department and neurology department with more than 5 years of clinical experience.

### Sample size

This clinical trial was a small sample pre-test, with a minimum sample size of 10–15 cases per group as recommended in the literature (21). Taking into account objective factors such as treatment duration, research funding limitations, and number of patients, the final sample size for this trial was determined to be 20 cases per group.

### Data processing and statistics

This experiment collected data through a central random system, established a terminal database, and used SPSS 22.0 software for data analysis and statistical description. For quantitative data, normal analysis and homogeneity of variance test were used. When both conditions were met, paired sample t-test was selected for intra group data, and independent sample t-test was selected for inter group data; If only a normal distribution was satisfied, the corrected t’ test was used; If neither of the above two conditions were met, then the rank sum test was chosen; Quantitative data that satisfied normality is represented by mean ± standard deviation ( $\bar{X}\pm S)$ ; When not satisfied, the median [M (P25, P75)] was used to represent. Qualitative data selection chi square test was expressed in percentage (%); Rank sum test was used for selecting grade data. All tests were conducted on both sides, with *p* < 0.05 indicated statistical significance.

## Results

### Descriptive data

**Figure 4:**
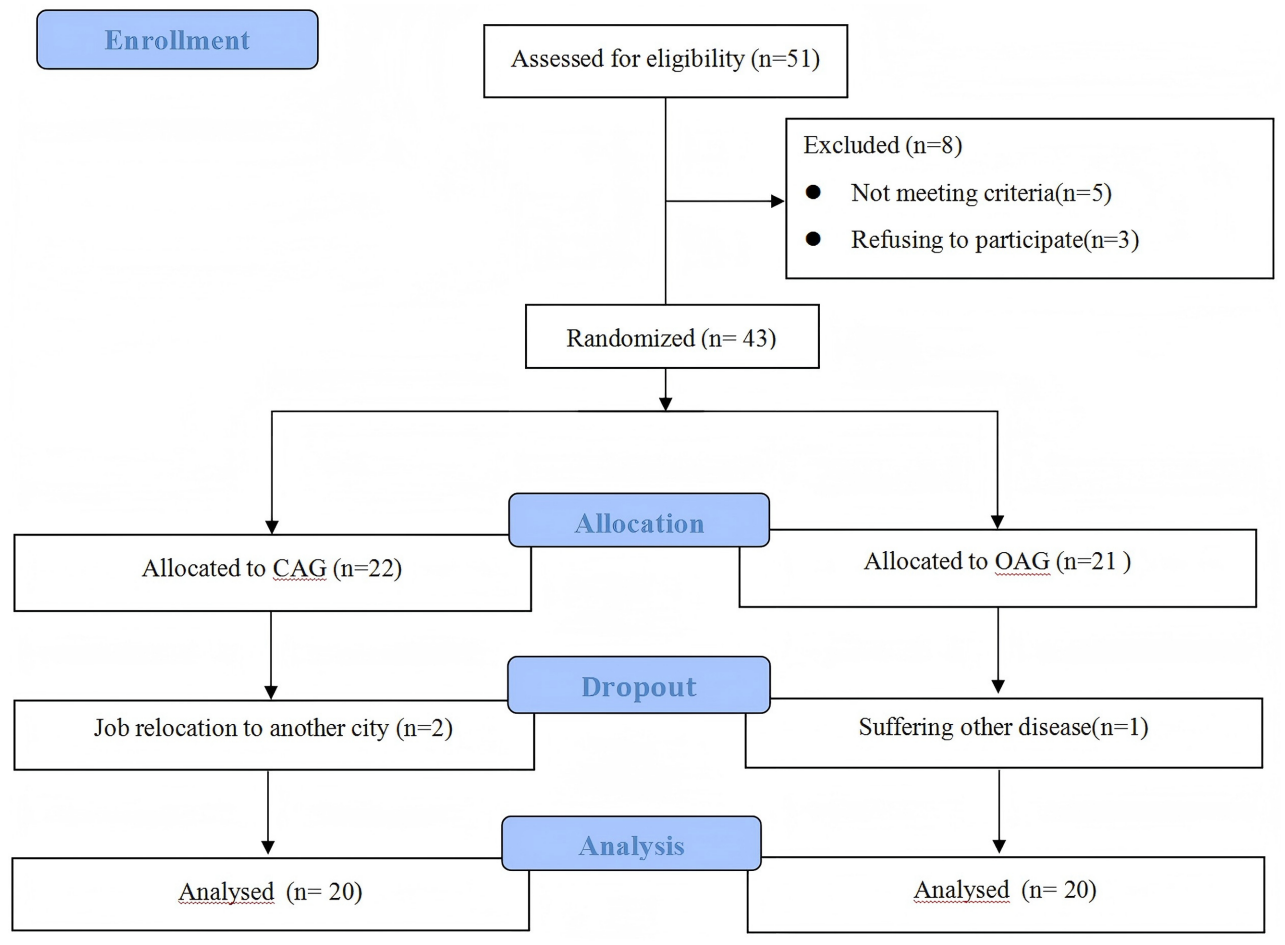
Patients progress thoughts the trial.

All participants in this study provided written informed consent Patients progress thoughts the trial: CONSORT flowchart was illustrated in Figure 4. A non-probability convenience sampling method was employed, and the data analysis adhered to per-protocol principles. We enrolled 51 patients for evaluation; however, 8 were excluded from the study (5 did not meet the inclusion criteria, and 3 refused to participate). As a result, 43 patients participated in the trial, and during the treatment period, 3 patients dropped out (2 patients from the CAG due to job relocation to another city, 1 patient from the OAG suffered other disease). Finally, a total of 40 IFP patients completed a three course study. The CAG group (*n* = 20) comprised 7 males and 13 females, with an average age of (46.10 ± 14.45) years and a disease course of (3.64 ± 0.48) months, with left-side of the affected face involvement in 14 cases and right-side in 6 cases. The OAG group (*n* = 20) comprised 11 males and 9 females, with an average age of (51.60 ± 14.00) years and a disease course of (3.73 ± 0.57) months, with left-side of the affected face involvement in 13 cases and right-side in 7 cases. There were no statistical significance between the two groups in terms of gender, age, disease course and affected side, as shown in Table 1.

**Table 1:** Comparison of general data of the two groups.

Parameter	CAG (*n* = 20)	OAG (*n* = 20)	X^2^/t	*p* value
Gender (male/female)	7/13	11/9	1.62	0.20^a^
Average age, age	46.10 ± 14.45*	51.60 ± 14.00*	1.22	0.23^b^
Disease course, month	3.64 ± 0.48*	3.73 ± 0.57*	1.34	0.19^a^
Affected side, left/right	14/6	13/7	0.11	0.74^b^

CAG = characteristic acupuncture group; OAG = ordinary acupuncture group.

*The data is presented in the form of mean ± standard deviation.

^a^Chi square test was used; ^b^Two independent sample t-tests was used.

### Anzhong Facial Paralysis Precision Scale (Oral Commissure Ptosis Grading Scale)

There was no statistical significance in the Anzhong Facial Paralysis Precision Scale (Oral Commissure Ptosis Grading Scale) levels between the two groups before treatment (*p* > 0.05), indicating comparability. After the first treatment course, there was no statistical significance (*p*>0.05) between the two groups. After the second treatment course, the difference between the two groups began to show statistical significance (*p* < 0.05), the CAG group demonstrated superior therapeutic efficacy compared to the OAG group, as shown in Table 2.

**Table 2:** Intergroup comparison of Anzhong Facial Paralysis Precision Scale (Oral Commissure Ptosis Grading Scale) levels across treatment courses.

Group	Level 0 (example)	Level 1 (example)	Level 2 (example)	Level 3 (example)	Total
Before treatment					
CAG	3	9	7	1	20
OAG	2	8	8	2	20
*Z* = −0.76, *p* = 0.45					
After the first treatment course					
CAG	8	7	5	0	20
OAG	4	9	6	1	20
*Z* = −1.28, *p* = 0.202					
After the second treatment course					
CAG	12	6	2	0	20
OAG	6	8	5	1	20
*Z* = −2.07, *p* = 0.038					
After the third treatment course					
CAG	14	5	1	0	20
OAG	7	9	3	1	20
*Z* = −2.28, *p* = 0.023					

CAG = characteristic acupuncture group; OAG = ordinary acupuncture group.

Rank sum test was used for grade data.

### SFGS scale scores

There was no statistical significance in SFGS scale scores between the two groups before treatment (*p* > 0.05), indicating comparability. After the first and second treatment courses, there was no statistical significance in scores changes between the two groups (*p* > 0.05). After the third treatment course, the scores changes between the two groups began to show statistical significance (*p* < 0.05), the CAG group demonstrated superior therapeutic efficacy compared to the OAG group, as shown in Table 3.

**Table 3:** Intergroup comparison of SFGS scale scores (*X– ± S*) across treatment courses.

Group	Cases	Before treatment	After the first treatment course	After the second treatment course	After the third treatment course
CAG	20	29.20 ± 17.19	41.01 ± 18.53	54.31 ± 21.03	65.60 ± 18.12
OAG		36.30 ± 15.24	48.90 ± 17.28	61.25 ± 18.21	69.20 ± 16.42
Scores changes in CAG compared to previous treatment			11.80 ± 6.72	13.30 ± 6.26	11.30 ± 4.43
Scores changes in OAG compared to previous treatment			12.60 ± 6.74	12.35 ± 6.52	7.95 ± 4.26
T value/χ^2^		−1.38	−0.38	0.47	2.44
*p* value		0.17	0.71	0.64	0.02

SFGS=Sunnybrook Facial Grading System; CAG = characteristic acupuncture group; OAG = ordinary acupuncture group.

Inter group comparison used two independent sample t-test.

### RMS average ratio

There was no statistical significance (*p* > 0.05) in the RMS average ratio of sEMG on the affected and healthy sides of the 5 acupoints between the two groups before treatment, indicating comparability. After the first treatment course, there was statistical significance in the average ratio changes at LI20 (Yingxiang) and ST4 (Dicang) between the two groups (*p* < 0.05). After the second treatment course, statistical significance in the average ratio changes was observed at GB14 (Yangbai), SI18 (Quanliao), LI20 (Yingxiang), and ST4 (Dicang) between the two groups (*p* < 0.05). After the third treatment course, statistical significance in the average ratio changes was found at all acupoints between the two groups (*p* < 0.05). Consequently, the CAG group demonstrated superior therapeutic efficacy compared to the OAG group, as shown in Table 4.

**Table 4:** Intergroup comparison of RMS average ratio (*X– ± S*) on the affected and the healthy sides across treatment courses.

Group	Cases	GB14 (Yangbai)	SI18 (Quanliao)	ST6 (Jiache)	LI20 (Yingxiang)	ST4 (Dicang)
Before treatment						
CAG	20	0.44 ± 0.15	0.36 ± 0.08	0.26 ± 0.19	0.35 ± 0.18	0.43 ± 0.23
OAG		0.38 ± 0.14	0.33 ± 0.14	0.28 ± 0.17	0.29 ± 0.20	0.41 ± 0.14
T value/χ^2^		1.33	0.94	−0.45	0.85	0.26
*p* value		0.19	0.35	0.65	0.40	0.79
After the first treatment course						
CAG	20	0.50 ± 0.14	0.44 ± 0.09	0.40 ± 0.20	0.45 ± 0.17	0.52 ± 0.22
OAG		0.41 ± 0.15	0.39 ± 0.12	0.39 ± 0.23	0.36 ± 0.21	0.47 ± 0.13
Average ratio changes in CAG compared to before treatment		0.07 ± 0.03	0.08 ± 0.06	0.13 ± 0.06	0.10 ± 0.06	0.09 ± 0.02
Average ratio changes in OAG compared to before treatment		0.04 ± 0.06	0.06 ± 0.04	0.10 ± 0.08	0.06 ± 0.05	0.05 ± 0.05
T value/χ^2^		1.79	1.30	1.56	2.12	2.70
*p* value		0.08	0.20	0.13	0.04	0.01
After the second treatment course						
CAG	20	0.59 ± 0.13	0.56 ± 0.12	0.44 ± 0.21	0.59 ± 0.15	0.63 ± 0.20
OAG		0.45 ± 0.16	0.45 ± 0.14	0.42 ± 0.23	0.42 ± 0.24	0.54 ± 0.13
Average ratio changes in CAG compared to the first treatment		0.08 ± 0.05	0.11 ± 0.05	0.05 ± 0.05	0.14 ± 0.06	0.11 ± 0.06
Average ratio changes in OAG compared to the first treatment		0.04 ± 0.07	0.06 ± 0.05	0.03 ± 0.01	0.06 ± 0.03	0.07 ± 0.03
T value/χ^2^		2.36	3.18	1.62	4.81	2.28
*p* value		0.02	0.03	0.11	<0.001	0.03
After the third treatment course						
CAG	20	0.67 ± 0.12	0.63 ± 0.11	0.51 ± 0.22	0.69 ± 0.13	0.72 ± 0.17
OAG		0.51 ± 0.16	0.51 ± 0.15	0.44 ± 0.023	0.49 ± 0.25	0.61 ± 0.12
Average ratio changes in CAG compared to the second treatment		0.07 ± 0.03	0.07 ± 0.02	0.06 ± 0.02	0.10 ± 0.04	0.09 ± 0.04
Average ratio changes in OAG compared to the second treatment		0.05 ± 0.03	0.06 ± 0.03	0.02 ± 0.01	0.07 ± 0.04	0.06 ± 0.03
T value/χ^2^		2.26	2.04	8.32	2.13	2.32
*p* value		0.03	0.04	<0.001	0.03	0.02

RMS = root mean square; CAG = characteristic acupuncture group; OAG = ordinary acupuncture group.

Inter group comparison used two independent sample t-test.

### Adverse events

No adverse reactions or safety incidents occurred during the research process.

## Discussion

This study was a randomized controlled pilot trial exploring the clinical efficacy of acupuncture and acupuncture manipulation in treating IFP, and observing their effectiveness and safety. The research results could be used to improve the diagnosis and treatment methods of IFP.

### Test results

In this study, the differences in the Anzhong Facial Paralysis Precision Scale (Oral Commissure Ptosis Grading Scale) levels between the two groups began to show statistical significance after the second treatment course (*p* < 0.05); The scores changes in SFGS scale between the two groups began to show statistical significance after the third treatment course (*p* < 0.05); After the first treatment course, there was statistical significance in the RMS average ratio changes at two acupoints between the two groups. After the second treatment course, it increased to four acupoints, and after the third treatment course, all acupoints showed statistical significance. Consequently, the CAG group demonstrated superior therapeutic efficacy compared to the OAG group.

### Acupuncture manipulation

From the results of this study, it can be seen that the changes in observation indicators in the OAG were slower than those in the CAG. After three treatment courses, the values showed an overall upward trend, and the improvement in the later stage of treatment was smaller. The reason may be that ordinary acupuncture caused non inflammatory exudation, muscle hardening, muscle adhesion and other symptoms in facial tissues, and even “inversion” symptoms, which caused the nerves on the affected side that should have been excited to be suppressed, aggravated the condition, and reduced the efficacy of acupuncture. The observation index values of the CAG showed a significant and stable change, indicating that the characteristic acupuncture manipulation has a mitigating effect on the above situation. Acupuncture manipulation is a key factor that directly affects the efficacy of acupuncture. Studies have shown that acupuncture manipulation has a significant effect on IFP (22), which can speed up the repair of facial nerves. Therefore, in the treatment, it is necessary to avoid the “wrong direction” growth of nerve fibers regenerated during nerve repair, and pay attention to reducing the possible damage to the body during treatment, so as to avoid adhesion during the repair process, which may cause facial muscle linkage reaction, and even lead to facial muscle atrophy or hyperplasia (23).

### Observation indicators

Previous clinical studies on peripheral facial paralysis have mostly used paper quality scales for evaluating therapeutic effects, but the scales have shortcomings such as imprecise local scoring and grading, difficulty in reflecting subtle changes in efficacy evaluation, poor sensitivity, strong subjectivity, poor targeting, and insufficient consideration of sequelae. The Anzhong Facial Paralysis Precision Scale is a refined facial paralysis evaluation scale system developed by the team based on neuroanatomy and clinical imaging. Following clinical evaluation scale setting rules and expert discussions, the branches and related symptoms of each facial nerve are defined, forming four dimensions: Eyebrow Ptosis Grading Scale, Eyelid Closure Grading Scale, Oral Commissure Ptosis Grading Scale and Philtrum Deviation Grading Scale. Each dimension has six entries. Fine differentiation of the 5 branches of the extracranial segment of the facial nerve entering the parotid gland, including the temporal branch: innervating the frontal and orbicularis oculi muscles; Zygomatic branch: innervates the orbicularis oculi muscle and zygomatic muscle; Buccal branch: innervates the cheek muscles, orbicularis oris muscles, and other perioral muscles; Mandibular marginal branch: distributed in the muscles of the lower lip; Neck branch: innervates the latissimus muscle of the neck. This study used the Anzhong Facial Paralysis Precision Scale (Oral Commissure Ptosis Grading Scale) to evaluate the damage and recovery of the buccal branch after facial paralysis. It can objectively and finely measure and evaluate the severity of clinical symptoms and recovery degree of facial paralysis by observing the relevant symptoms in the affected areas caused by damage to different branches during facial paralysis, and grading the severity of symptoms in corresponding parts of different branches. To compensate for the shortcomings of the paper quality chart, this study also used advanced sEMG technology as a detection method for post-treatment evaluation. sEMG is mainly used in muscle function evaluation and fatigue assessment in the rehabilitation field, and has advantages in evaluating compensatory movements after chronic facial nerve disease or facial nerve paralysis. It is an important neurophysiological diagnostic tool for evaluating facial nerve function and detecting nerve regeneration (24), which can objectively compare clinical efficacy. The observation indicators of this study have both subjective and objective observation indicators, and the combination of the two can be more complementary, evaluating the efficacy from multiple perspectives, reducing the occurrence of potential bias, and thus improving the quality of clinical evidence.

This study observed the evaluation effect of the Anzhong Facial Paralysis Precision Scale (Oral Commissure Ptosis Grading Scale) on the function of the facial nerve buccal branch innervated area, namely the orbicularis oris and cheek muscles in IFP patients. The innovation of this scale lies in using the oral commissure on the healthy side of IFP patients as the measurement benchmark, which can greatly avoid the interference caused by facial asymmetry of patients themselves, and can more objectively and directly observe whether patients have “inversion” phenomenon. Based on the comprehensive comparison of the two scales and sEMG, the SFGS scale was used to calculate the dynamic and static scores of facial paralysis. Statistical significance in scores changes between the two groups were only evident after the third treatment course. In contrast, the Anzhong Facial Paralysis Precision Scale (Oral Commissure Ptosis Grading Scale) levels showed statistical significance after the second treatment course. The sEMG results indicated that only one acupoint’s RMS average ratio changes between the two groups did not show statistical significance after the second treatment course while statistical significance was observed at the LI20 (Yingxiang) and ST4 (Dicang) acupoints after the first treatment course. Relatively speaking, the sEMG results were more consistent with the findings from the Anzhong Facial Paralysis Precision Scale (Oral Commissure Ptosis Grading Scale) levels. Current literature indicates that clinical trials investigating acupuncture treatment for IFP often require more than 20 times (25, 26) to observe positive changes in the outcome measures, with some trials exceeding 30 times (27, 28). This information is largely consistent with the findings of the present study. This indicates that the Anzhong Facial Paralysis Precision Scale (Oral Commissure Ptosis Grading Scale) is more precise than the SFGS scale in evaluating the recovery of the oral commissure, and its combination with sEMG can be incorporated into the refined evaluation system of facial paralysis neuromuscular function as an objective and reliable diagnostic basis for clinical promotion and use.

## Limitations

Despite the significant improvements observed in the efficacy of acupuncture for treating IFP in this study, several limitations remain: (1) this is a single-center study with a relatively small sample size, which may affect the statistical significance of the findings. The single-center design may also introduce selection bias, both of which impact the generalizability of the results; (2) due to the nature of the intervention, acupuncturists were not blinded to treatment conditions, which could introduce potential bias; (3) in chronic or progressive diseases, short-term outcomes may differ significantly from long-term effects. The absence of follow-up assessments in this study limits our ability to evaluate the durability of the results.

In future research, measures will be implemented to blind participants, data collectors, outcome assessors, and data analysts. A multi-center, large-sample clinical trial will be conducted, including subgroup analyses based on factors such as age, disease duration, and overall health status. Follow-up assessments will also be integrated. These strategies will effectively reduce bias in clinical trials, enhance the validity and reliability of research findings, and facilitate the translation of clinical research outcomes into reliable clinical practice.

## Conclusion

This pilot trial provides a preliminary exploration of the clinical efficacy of acupuncture and manual therapy for treating IFP. The results indicate that acupuncture may offer a certain therapeutic benefit for IFP, and the acupuncture intervention plays a significant role in improving treatment outcomes. Furthermore, Anzhong Facial Paralysis Precision Scale (Oral Commissure Ptosis Grading Scale) showed advantages in sensitivity when assessing patients’ recovery of facial nerve function. However, these conclusions require further validation in larger clinical studies. This outcome offers initial guidance for the treatment and evaluation of facial paralysis patients in later research.

## Data Availability

The raw data supporting the conclusions of this article will be made available by the authors, without undue reservation.
